# A Novel Nomogram Integrating Retinal Microvasculature and Clinical Indicators for Individualized Prediction of Early Neurological Deterioration in Single Subcortical Infarction

**DOI:** 10.1111/cns.70337

**Published:** 2025-03-12

**Authors:** Chen Ye, William Robert Kwapong, Le Cao, Hui Xu, Yanan Wang, Yuying Yan, Ruosu Pan, Ruilin Wang, Kun Lu, Lanhua Liao, Tang Yang, Shuai Jiang, Xuening Zhang, Wendan Tao, Junfeng Liu, Bo Wu

**Affiliations:** ^1^ Department of Neurology West China Hospital, Sichuan University Chengdu China; ^2^ Center of Cerebrovascular Diseases West China Hospital, Sichuan University Chengdu China; ^3^ Department of Neurology Xuanwu Hospital, Capital Medical University Beijing China; ^4^ Department of Radiology West China Hospital, Sichuan University Chengdu China; ^5^ Department of Ophthalmology West China Hospital, Sichuan University Chengdu China

**Keywords:** early neurological deterioration, nomogram, prediction, retinal microvasculature, subcortical infarction

## Abstract

**Aims:**

Early neurological deterioration (END) is a relatively common occurrence among patients with single subcortical infarctions (SSI). Accurate and early prediction of END in SSI is challenging and could contribute to enhancing prognosis.

**Methods:**

This prospective observational study enrolled SSI patients who arrived within 24 h from symptom onset at a single center between December 2020 and March 2023. The least absolute shrinkage and selection operator (LASSO) regression model was applied to optimize feature selection for the predictive model. A nomogram was generated based on multivariate logistic regression analysis to identify potential predictors associated with the risk of END. The performance and clinical utility of the nomogram were generated using Harrell's concordance index, calibration curve, and decision curve analysis (DCA).

**Results:**

Of 166 acute SSI patients, 45 patients (27.1%) developed END after admission. The appearance of END is associated with four routine clinical factors (NIHSS score, serum neuron‐specific enolase, uric acid, periventricular white matter hyperintensity), and two retinal microvascular indicators (ipsilateral superficial and deep vascular complexes). Incorporating these factors, the nomogram model achieved a concordance index of 0.922 (95% CI 0.879–0.964) and had a well‐fitted calibration curve and good clinical application value by DCA. A cutoff value of 203 was determined to predict END via this nomogram.

**Conclusions:**

This novel nomogram exhibits high accuracy in predicting END in SSI patients. It could guide clinicians to identify SSI patients with a high risk of END at an early stage and initiate necessary medical interventions, ultimately leading to a better prognosis.

## Graphical abstract

We developed a novel nomogram that incorporated four routine clinical variables with two OCTA‐derived retinal microvascular metrics in predicting END in SSI patients, which exhibits high accuracy in predicting END in SSI patients. It could help identify SSI patients with a high risk of END at an early stage and benefit a better prognosis.

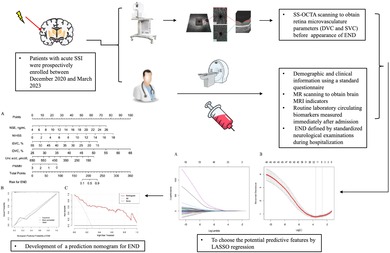

## 1 Introduction

Single subcortical infarction (SSI) constitutes about a quarter of all ischemic stroke subtypes, which are usually thought to be mildly symptomatic and have a favorable outcome [1, 2]. Nevertheless, over 20% of patients with SSI suffer from early neurological deterioration (END), which results in more severe neurological deficits. The etiology and pathogenesis of END in SSI are complex, and clear descriptions, accurate and reliable early prediction indicators, and effective prevention and treatment strategies are lacking [1, 3, 4].

The cause of SSI involves macrovascular and microvascular causes [5]. There is considerable homology in the retinal and cerebral microcirculation, and retinal microvascular changes may reflect changes in the cerebral microcirculation. This concept is supported by clinical studies showing a range of retinal microvascular changes in SSI patients, such as vessel caliber and retinopathy signs [6, 7]. Using the swept‐source optical coherence tomography angiography (SS‐OCTA), it has been shown that SSI patients have sparser retinal microvasculature compared to controls [8]. Besides, retinal changes were found to be associated with SSI patients' neurological deficit, which was measured by the National Institute of Health Stroke Scale (NIHSS) score [9]. Recent studies using retinal imaging modalities showed that sparser retinal microvasculature can be used to predict the incidence of stroke mortality in older persons [10, 11]. These reports demonstrated that retinal microvasculature reflects cerebral microcirculation, and changes in the retinal microvasculature reflect neurological damage in SSI patients. Importantly, retinal imaging with OCTA has the potential to be used as a tool to detect microvascular damage in patients with stroke.

Using the retinal microvasculature as a proxy to the cerebral microcirculation, we aimed to develop an accurate and novel prediction nomogram that predicts END in SSI patients by combining their clinical and radiological information with OCTA‐derived retinal microvasculature parameters.

## 2 Methods

### 2.1 Study Design and Participants

This observational‐cohort study was conducted at the Department of Neurology, West China Hospital of Sichuan University, and was approved by the Ethics Committee of West China Hospital, Sichuan University [No. 2020 (922)] and followed the Declaration of Helsinki, reported according to the TRIPOD (Transparent reporting of a multivariable prediction model for individual prognosis or diagnosis) guideline (https://www.equator‐network.org/reporting‐guidelines/tripod‐statement/). All participants or legal guardians signed an informed consent before enrolling in our study. Between December 2020 and March 2023, we prospectively enrolled acute ischemic stroke patients with a suspected diagnosis of SSI [12] admitted to the Department of Neurology, West China Hospital. The general inclusion criteria and exclusion criteria can be found in a previous report [8]. Given that the objective of this study is to develop a predictive model for the occurrence of END, our research team collaborated with the Departments of Neurology, Radiology, and Ophthalmology to ensure that blood sample collection, MRI examinations, and fundus SS‐OCT/OCTA image acquisitions were all completed prior to the onset of END for all included patients.

### 2.2 Clinical Data Collection

Demographic and clinical information, including age, gender, vascular risk factors (hypertension, diabetes mellitus, atrial fibrillation, hyperlipidemia, smoking, and drinking), body mass index (BMI), National Institute of Health Stroke Scale (NIHSS) score on admission, and therapies after admission (mono‐ or dual‐antiplatelets, thrombolysis, antidiabetic, antihypertension, and lipid‐lowering) were collected using a standard questionnaire. Systolic blood pressure (SBP) and diastolic blood pressure (DBP) were measured and obtained at admission. Laboratory blood circulating biomarkers were obtained and selected based on previous reports [13, 14, 15, 16, 17, 18, 19, 20, 21, 22, 23]. Accordingly, 22 biomarkers indicating coagulation disorder [red cells, platelets, and fibrinogen (FIB)], inflammation [white cells, neutrophils, lymphocytes, monocytes, neutrophil to lymphocyte ratio (N‐L‐R), lymphocyte to monocyte ratio (L‐M‐R), and systemic immune‐inflammation index (S‐I‐I)], lipid metabolism [triglyceride (TG), cholesterol, high‐density lipoprotein (HDL), and low‐density lipoprotein (LDL)], liver function [alkaline phosphatase (ALP), alanine aminotransferase (ALT), aspartate aminotransferase (AST), and total bilirubin (TB)], vasoreactivity (creatinine), neuroprotection and neural injury [uric acid and neuron‐specific enolase (NSE)], and fast blood glucose were measured immediately after admission.

### 2.3 Brain Imaging Data Collection

**FIGURE 1:**
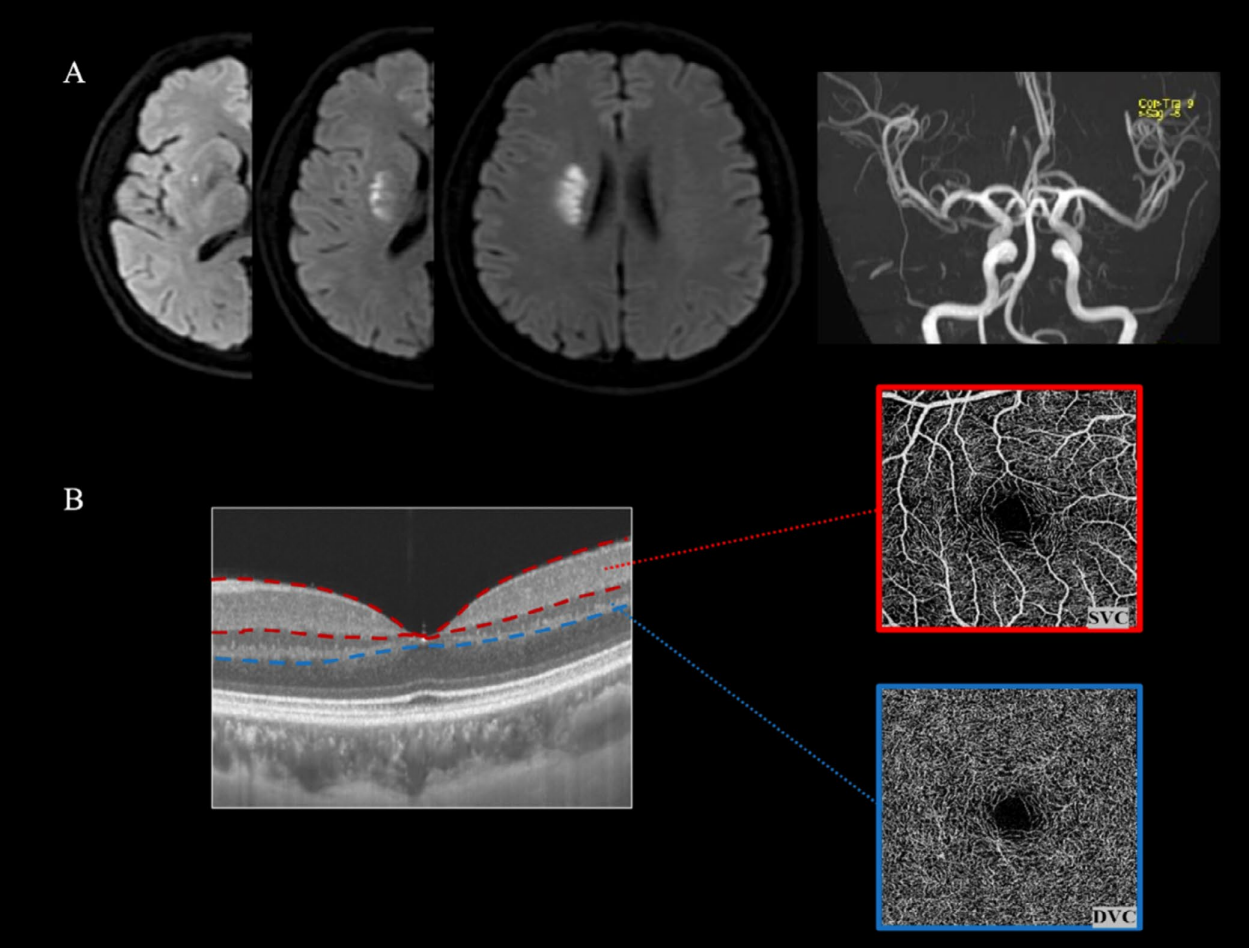
Illustrations of lesion characteristics measurements and retinal microvasculature segmentations. (A) Axial diffusion‐weighted imaging of one patient in our cohort showed an acute subcortical infarct lesion in the right basal ganglia on three slices, and an intracranial artery reconstruction image using conventional magnetic resonance angiography showed no stenosis in the relevant middle cerebral artery (MCA). (B) En face angiography for superficial vascular complex (SVC) and deep vascular complex (DVC). The segmentation between the SVC and the DVC was set at the inner two‐thirds and outer one‐third interface of the ganglion cell layer and inner plexiform layer.

All enrolled participants underwent 3.0 Tesla brain magnetic resonance imaging (MRI) within 24 h after admission. The MRI protocol included DWI, magnetic resonance angiography (MRA), T1‐weighted, T2‐weighted, and fluid‐attenuated inversion recovery (FLAIR) images. As shown in Figure 1A, the axial maximum diameter of the infarct lesion on DWI and the total slices of infarct planes were obtained. Details of the MRI protocol and analysis procedure are well detailed in our previous reports [1, 5]. Based on the perforator territory patterns [24], the lesion locations were further classified into basal ganglia, thalamus, centrum semiovale, and brainstem.

The severity of white matter hyperintensities (WMH) was defined on the FLAIR sequence using Fazekas scores [25]. Periventricular (PWMH), deep WMH (DWMH), and total WMH scores were recorded. Symptomatic intracranial hemorrhage (sICH) was defined according to the National Institute of Neurological Diseases and Stroke Study (NINDS) criteria [26].

Two trained neurologists (C.Y. and R.P.) were engaged in the MRI processing and measurements blinded to clinical information, and an experienced neurologist (B.W.) was consulted when disagreements occurred. The inter‐rater reliability of measurements for each neuroimaging marker mentioned above was considered good to excellent, as shown in the Supporting Information.

### 2.4 Retinal Microvascular Parameters Measurement by SS‐OCTA

The retinal microvascular parameters were measured by SS‐OCTA (SVision Imaging, Henan, China. Version 2.1.016) operated by an experienced neuro‐ophthalmologist (W.K.) within 24 h after admission. Notably, only patients with OCTA scanning examined before the appearance of END were enrolled in this study. OCTA fundus images were obtained at the macula with a raster scan protocol of 384 horizontal B‐scans that covered an area of 3 × 3 mm^2^ centered on the fovea. En‐face angiograms of the superficial vascular complex (SVC) and deep vascular complex (DVC) were generated by automatic segmentation, as shown in Figure 1B with detailed scanning descriptions presented in previous reports [27, 28, 29]. The percentage of the SVC and DVC was obtained with an in‐built algorithm in the OCTA tool in a 2.5 mm diameter circular region centered on the fovea.

Dr. Ruilin Wang, a specialized ophthalmologist, assessed all OCTA images. Angiograms with ophthalmic disorders such as age‐relatedmacular degeneration, severe cataracts, optic neuritis, retinal hemorrhage, diabetic retinopathy, glaucoma, and optic neuritis were excluded. If a participant presented with any of these disorders in one eye, the participant was excluded from the study. Angiograms with artifacts and a signal quality of less than 7 were also excluded from our study. The OCTA data displayed in our study followed the OSCAR‐IB quality criteria [30] and APOSTEL recommendation [31]. For this study, eyes were stratified as ipsilateral and contralateral to cerebral infarction.

### 2.5 Outcome

The early neurological deterioration (END) was defined as a ≥ 2 points increase in the total NIHSS score or a ≥ 1 point increase in the motor item of the NIHSS score compared with the best neurological status during the first 7 days of symptom onset after admission [4, 32]. Standardized neurological examinations, blinded to the collected clinical and imaging information, were conducted by two trained neurology residents (Y.Y. and C.Y.), and an experienced neurologist (B.W.) was consulted when disagreement occurred.

### 2.6 Statistical Analyses

The normality of the data was assessed by visual inspection of the distribution and the Kolmogorov–Smirnov test. Consecutive variables with normal distribution were expressed as mean and standard deviation (SD), while skewed distribution was expressed as median and interquartile ranges (IQR). Categorical variables were presented as frequencies and percentages (%). All statistical analyses were performed with R version 4.1.3. A two‐sided *p* < 0.05 was considered statistically significant.

A generalized estimating equation (GEE) was used to compare the differences in OCTA parameters in eyes ipsilateral and contralateral to infarction of SSI patients with and without END while adjusting for inter‐eye dependencies and vascular risk factors (age, gender, hypertension, diabetes mellitus, and hyperlipidemia).

To choose the potential predictive features from our study participants, we utilized the least absolute shrinkage and selection operator (LASSO) regression, which is an effective high‐dimensional prediction method. Meanwhile, the optimal value of λ was determined via fivefold cross‐validation. Then, we performed a multivariable logistic regression model to identify statistically significant predictors, which were then utilized to construct a nomogram. These features were presented as odds ratios (OR), 95% confidence intervals (95% CI), and *p*‐values.

To assess the overall discriminatory ability and calibration of the nomogram, the calibration was plotted. Meanwhile, the Hosmer–Lemeshow test was calculated (a significant statistic test indicated that the model is not well calibrated). Additionally, Harrell's concordance index (C‐index) and bootstrapping (1000 bootstrap replicates) were computed. Finally, to assess the clinical benefit of the END nomogram, we performed decision curve analysis (DCA) to measure the net benefits at threshold probabilities in our study cohort.

The cumulative scores for each patient were computed using the nomogram. The ideal threshold value, that is, the cutoff value, was identified by maximizing the Youden index (sensitivity + specificity—1) during receiver operating characteristic curve (ROC) analysis. Accuracy was evaluated through measures including sensitivity, specificity, predictive values, and likelihood ratios.

### 2.7 Sample Size

At present, the events per variable (EPV) criterion, particularly an EPV of 10, is commonly employed as the minimum threshold for logistic regression models aimed at predicting binary outcomes [33]. In this study, six variables were ultimately incorporated into the multivariable logistic regression analysis to forecast END. Consequently, the effective sample size for the derivation cohort should comprise a minimum of 60 patients. Therefore, the effective sample size of the derivation cohort should be at least 60 patients. Furthermore, given that the incidence of END in SSI is approximately 40% globally [1, 34], the final cohort should include at least 150 patients.

## 3 Results

### 3.1 Population Characteristic

**FIGURE 2:**
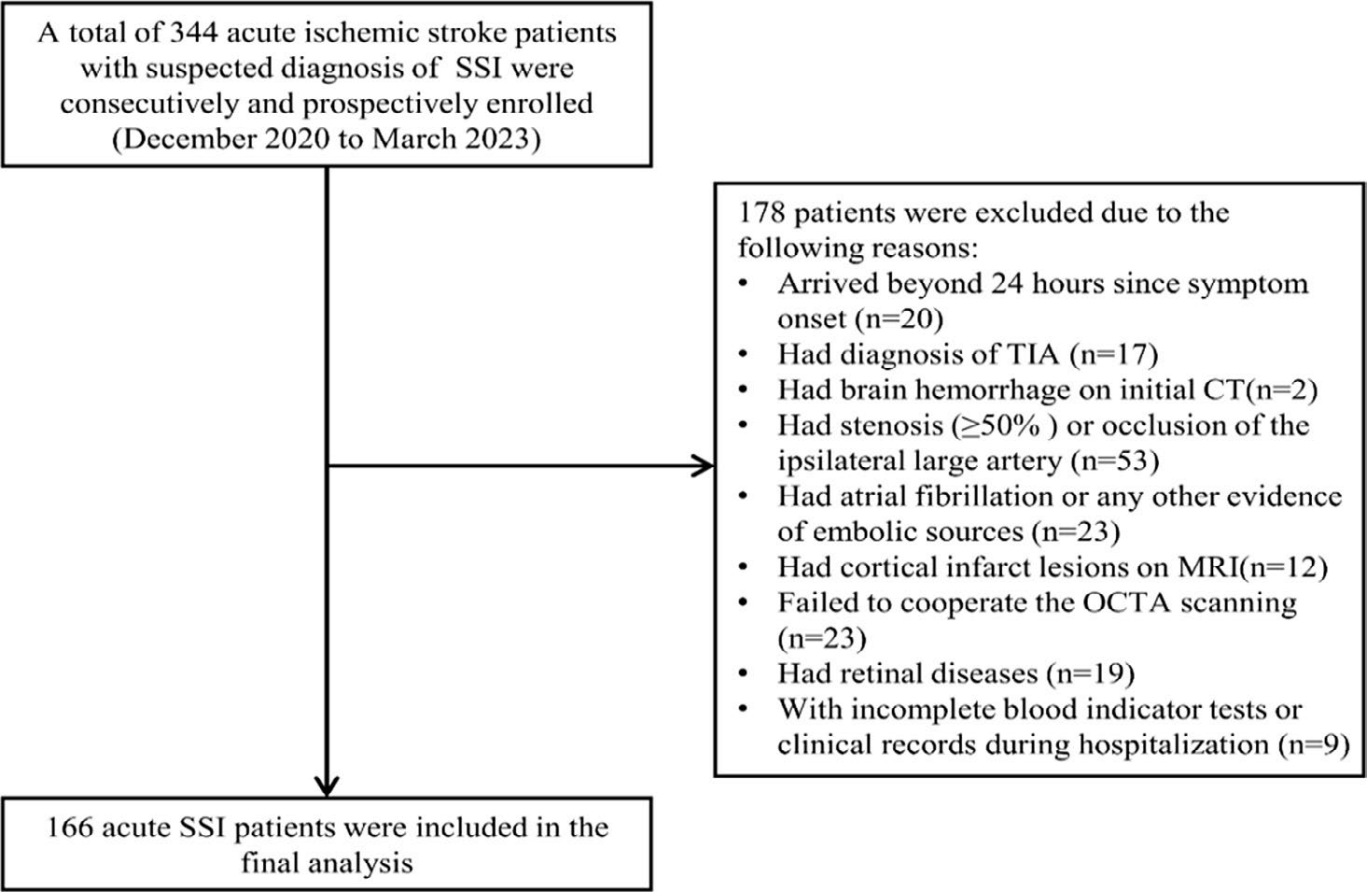
Flow chart of patient selection. A total of 344 acute ischemic stroke patients with a suspected diagnosis of SSI admitted to West China Hospital of Sichuan University were consecutively and prospectively enrolled from December 2020 to March 2023, of whom 178 were excluded due to being beyond 24 h since symptom onset (*n* = 20), had diagnosis of TIA (*n* = 17), have brain hemorrhage on initial CT (*n* = 2), have stenosis (≥ 50%) or occlusion of the ipsilateral large artery (*n* = 53), have atrial fibrillation or any other evidence of embolic sources (*n* = 23), have cortical infarct lesions on MRI (*n* = 12), fail to cooperate with the octa scanning (*n* = 23), have retinal diseases (*n* = 19), have incomplete blood indicator tests or clinical records during hospitalization (*n* = 9). The final analysis included 166 patients with acute SSI, of whom 45 (27.1%) developed early neurological deterioration (END).

**FIGURE 3:**
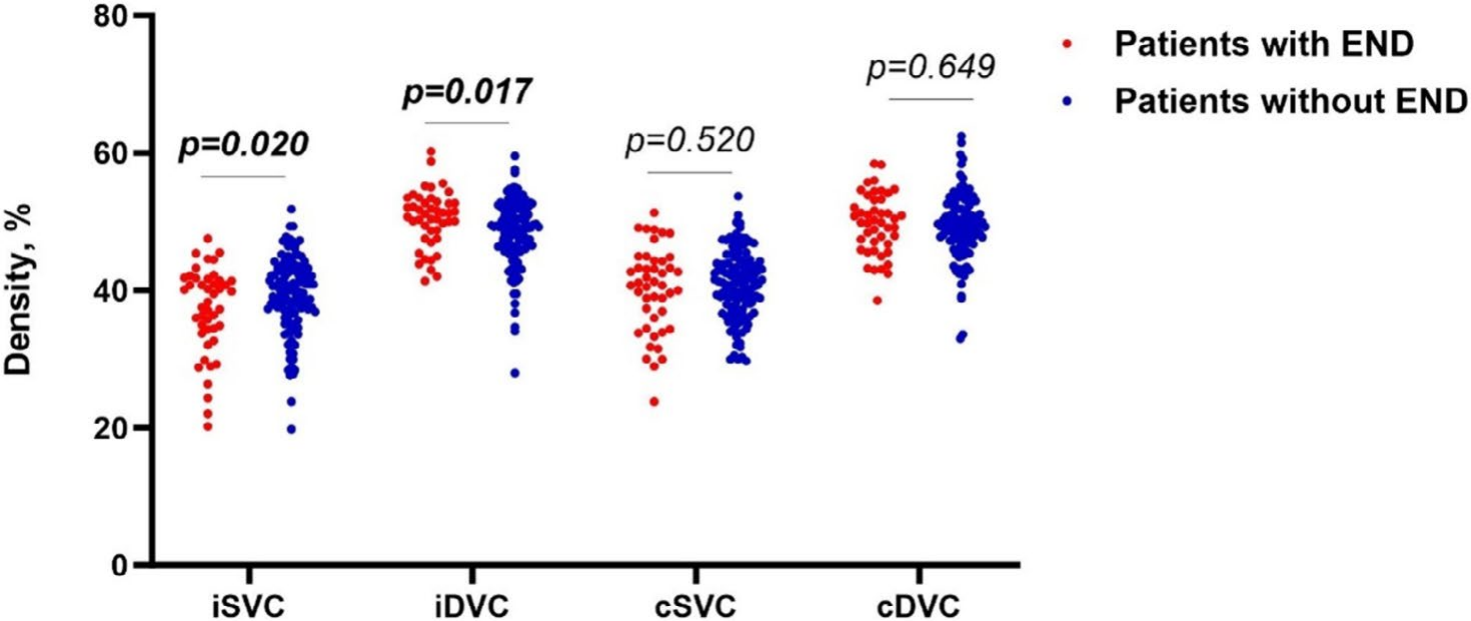
The comparison of OCTA parameters between SSI patients with and without END. Ipsilateral eyes of SSI patients with END showed lower SVC density (37.06 ± 6.35 vs. 39.39 ± 5.43, *p* = 0.020) and higher DVC density (50.42 ± 4.11 vs. 48.44 ± 4.93, *p* = 0.017) compared to contralateral eyes. In contralateral eyes, there were no significant differences in the SVC and DVC densities when both groups were compared. DVC, deep vascular complex; SVC, superficial vascular complex.

Figure 2 displays the inclusion and exclusion criteria of our study participants. Table 1 shows the demographics, clinical, radiological, and OCTA information of our study participants. One hundred sixty‐six SSI patients (mean age: 56.72 ± 10.40 years; 82.5% males) were included in our final data analysis. Of the 166 patients, 91 (54.8%) had an infarction in the basal ganglia, 34 (20.5%) in the thalamus, 27 (16.3%) in the centrum semiovale, while 14 (8.4%) in the brainstem. Importantly, 45 (27.1%) patients had END, while 121 patients were without END. Figure 3 shows the comparison of OCTA parameters between SSI patients with and without END. In ipsilateral eyes, patients with END showed lower SVC density (37.06 ± 6.35 vs. 39.39 ± 5.43, *p* = 0.020) and higher DVC (50.42 ± 4.11 vs. 48.44 ± 4.93, *p* = 0.017) compared to patients without END. In contralateral eyes, there were no significant differences in the SVC and DVC densities when both groups were compared. No significant difference was seen in the baseline characteristics when included and excluded participants were compared (Table S1).

**TABLE 1:** Baseline characteristics.

Variables	Included SSI patients (*n* = 166)
Demographic characteristics	
Age, years, mean ± SD	56.72 ± 10.40
Gender, male, *n* (%)	137 (82.5)
Clinical characteristics	
SBP on admission, mmHg, mean ± SD	144.35 ± 19.39
DBP on admission, mmHg, mean ± SD	88.62 ± 14.42
NIHSS on admission, median (IQR)	3 (1–5)
Vascular risk factors	
Current smoking, *n* (%)	86 (51.8)
Current alcohol use, *n* (%)	66 (39.8)
Hypertension, *n* (%)	102 (61.4)
Diabetes, *n* (%)	53 (31.9)
Dyslipidemia, *n* (%)	41 (24.7)
BMI, kg/m^2^, median (IQR)	24.32 (22.62–26.64)
Laboratory parameters on admission	
Red cell, ×10^12^/L, mean ± SD	4.80 ± 0.55
White cell, ×10^9^/L, mean ± SD	6.83 ± 1.95
Platelet, ×10^9^/L, mean ± SD	193.28 ± 64.83
Neutrophil, ×10^9^/L, median (IQR)	4.14 (3.29–5.38)
Lymphocyte, ×10^9^/L, median (IQR)	1.63 (1.26–1.99)
Monocyte, x 10^9^/L, median (IQR)	0.47 (0.40–0.60)
FIB, g/L, median (IQR)	2.74 (2.32–3.19)
N‐L‐R, median (IQR)	2.56 (1.92–3.23)
L‐M‐R, median (IQR)	3.35 (2.79–4.33)
S‐I‐I, ×10^9^/L, median (IQR)	479.67 (332.64–698.50)
Glucose, mmol/L, median (IQR)	6.48 (5.39–8.41)
TG, mmol/L, median (IQR)	1.52 (1.14–2.33)
Cholesterol, mmol/L, mean ± SD	4.35 ± 1.02
HDL, mmol/L, median (IQR)	1.07 (0.93–1.38)
LDL, mmol/L, mean ± SD	2.65 ± 0.87
ALT, IU/L, median (IQR)	22 (15–32)
AST, IU/L, median (IQR)	20 (17–25)
ALP, IU/L, median (IQR)	79 (68.5–98)
TB, μmol/L, median (IQR)	11.10 (8.70–14.35)
Creatinine, μmol/L, median (IQR)	75 (64–85.5)
Uric acid, μmol/L, mean ± SD	338.53 ± 91.79
Serum NSE, ng/mL, mean ± SD	12.14 ± 3.38
Treatment after admission, *n* (%)	
Mono antiplatelet	72 (43.4)
Dual antiplatelet	94 (56.6)
Thrombolysis	12 (7.2)
Antidiabetic	44 (26.5)
Antihypertension	97 (58.4)
Lipid lowering	163 (98.2)
Retinal microvascular parameters, mean ± SD	
SVC, %	44.08 ± 4.23
DVC, %	49.19 ± 4.35
Brain imaging parameters	
Axial maximal lesion diameter, mm, median (IQR)	15.5 (10.9–20.3)
Number of infarct slices, median (IQR)	3 (2–4)
PWMH, median (IQR)	1 (1–2)
DWMH, median (IQR)	1 (0–2)
Total WMH, median (IQR)	2 (1–3)
sICH, *n* (%)	3 (1.8)
Lesion site, *n* (%)	
Basal ganglia	91 (54.8)
Thalamus	34 (20 0.5)
Centrum semiovale	27 (16.3)
Brainstem	14 (8.4)
Outcome, *n* (%)	
END	45 (27.1)
Without END	121 (72.9)

*Note:* Data are *n* (%), mean (SD), or median (IQR).

Abbreviations: ALP, alkaline phosphatase; ALT, alanine aminotransferase; AST, aspartate aminotransferase; BMI, body mass index; DBP, diastolic blood pressure; DVC, deep vascular complex; DWMH, deep white matter hyperintensity; END, early neurological deterioration; FIB, fibrinogen; HDL, high‐density lipoprotein; LDL, low‐density lipoprotein; L‐M‐R, lymphocyte to monocyte ratio; N‐L‐R, neutrophil to lymphocyte ratio; NIHSS, National Institute of Health Stroke Scale score; NSE, neuron‐specific enolase; PWMH, periventricular white matter hyperintensity; SBP, systolic blood pressure; sICH, symptomatic intracranial hemorrhage; S‐I‐I, systemic immune‐inflammation index; SSI, single subcortical infarction; SVC, superficial vascular complex; TB, total bilirubin; TG, triglyceride.

**FIGURE 4:**
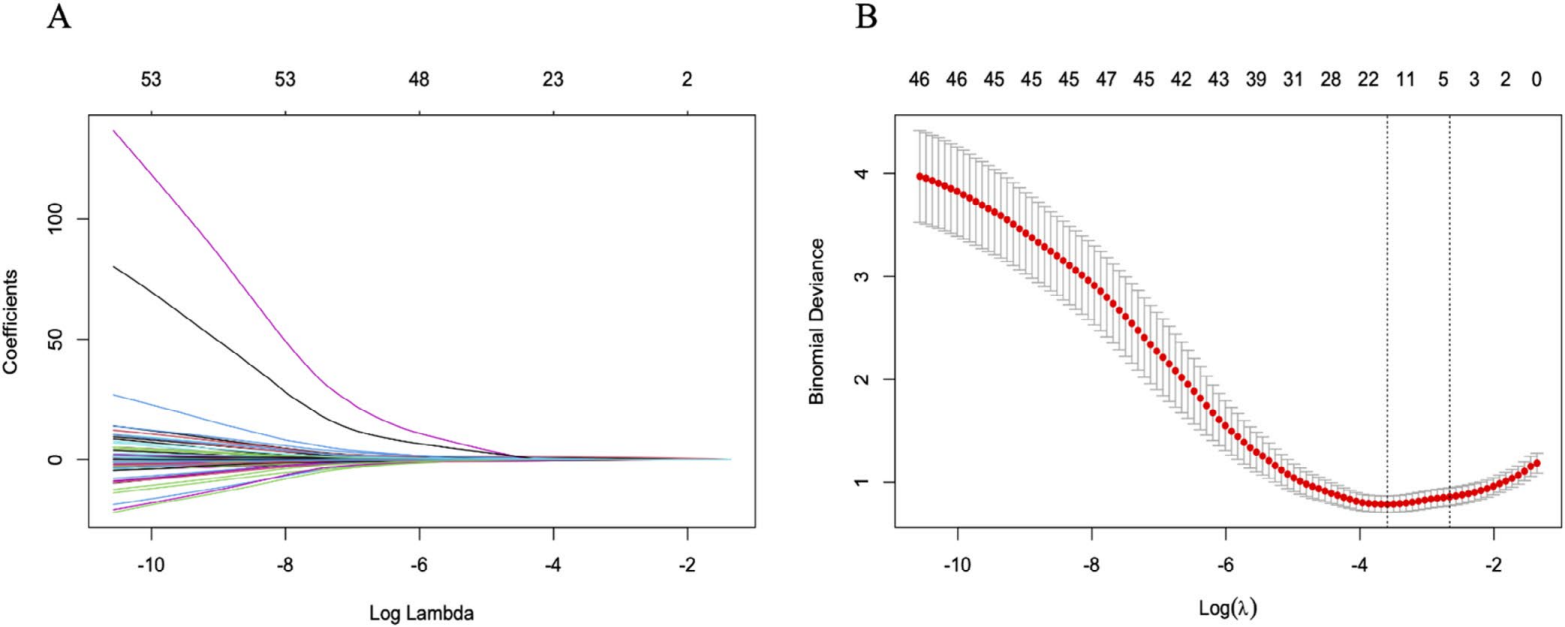
Feature selection using the LASSO binary logistic regression model. (A) Log (Lambda) value of the 14 features in the LASSO model. A coefficient profile was produced against the log (lambda) sequence. (B) Parameters selection in the LASSO model used fivefold cross‐validation via the minimum criterion. Partial likelihood deviation (binomial deviation) curves and logarithmic (lambda) curves were plotted. Use the minimum standard and 1se (1‐SE standard) of the minimum standard to draw a vertical dashed line at the optimal value. The optimal lambda produced four nonzero coefficients. LASSO, least absolute shrinkage and selection operator; SE, standard error.

Of the clinical information collected from our study participants, fourteen features were chosen based on the non‐zero coefficient calculated by LASSO logistic regression analysis (Figure 4A,B). The selected information included NSE, NIHSS, iSVC, iDVC, monocyte, ALP, serum uric acid, thrombolysis, anti‐diabetic, PWMH, number of infarct slices, infarction in basal ganglia, and sICH. These features were subsequently included in multivariate logistic regression analysis.

### 3.2 Development of Nomogram

**FIGURE 5:**
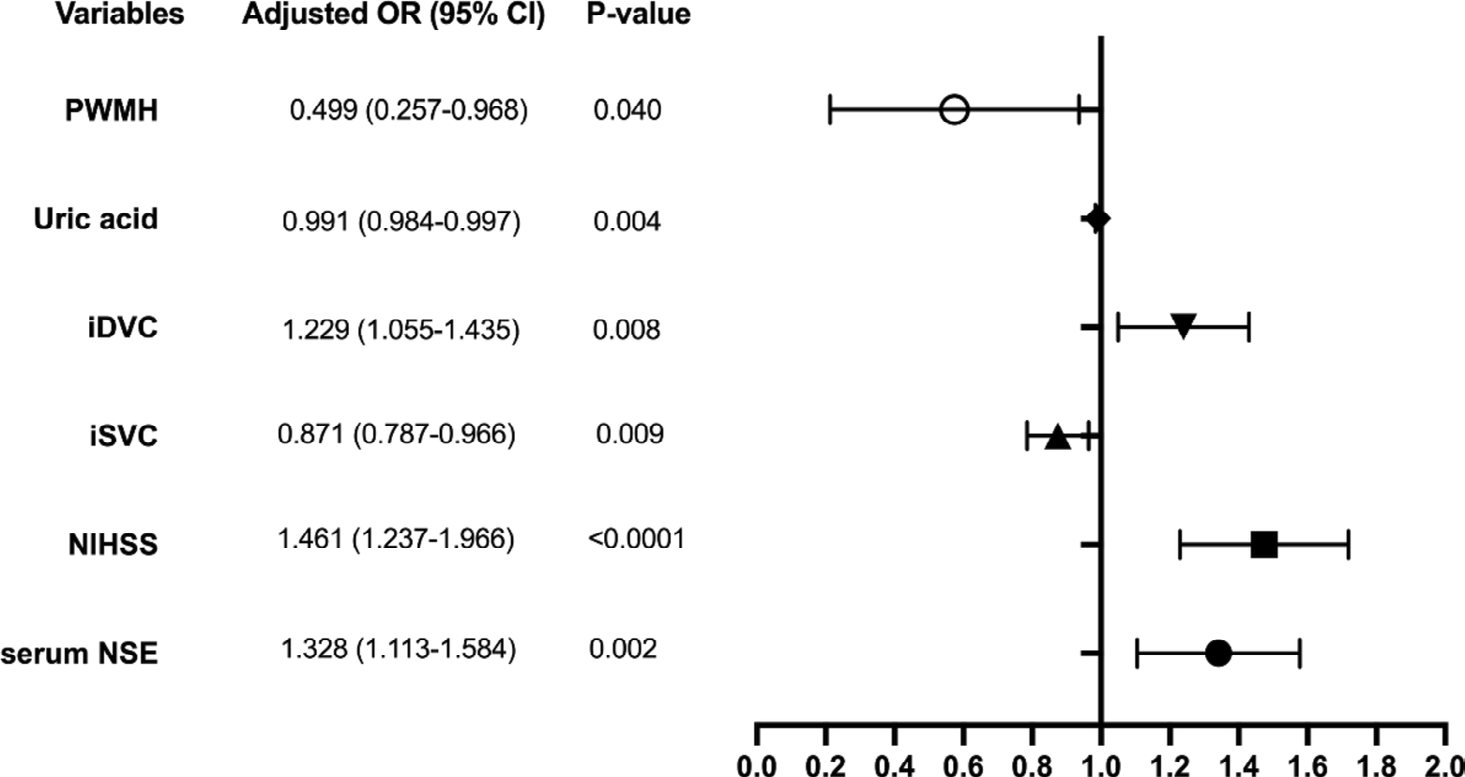
Multivariate logistic regression analyses and forest plots for assessing the predictors of END based on the features selected by the LASSO regression model. Based on the data obtained using LASSO analysis, the multivariate logistic regression analysis showed serum NSE (*p* = 0.002), NIHSS (*p* < 0.001), iSVC (*p* = 0.009), iDVC (*p* = 0.008), serum uric acid (*p* = 0.004) and PWMH (*p* = 0.040) were independently predictive for END. NIHSS, National Institute of Health Stroke Scale score. DVC, deep vascular complex; END, early neurological deterioration; NSE, neuron‐specific enolase; PWMH, periventricular white matter hyperintensity; SVC, superficial vascular complex.

**FIGURE 6:**
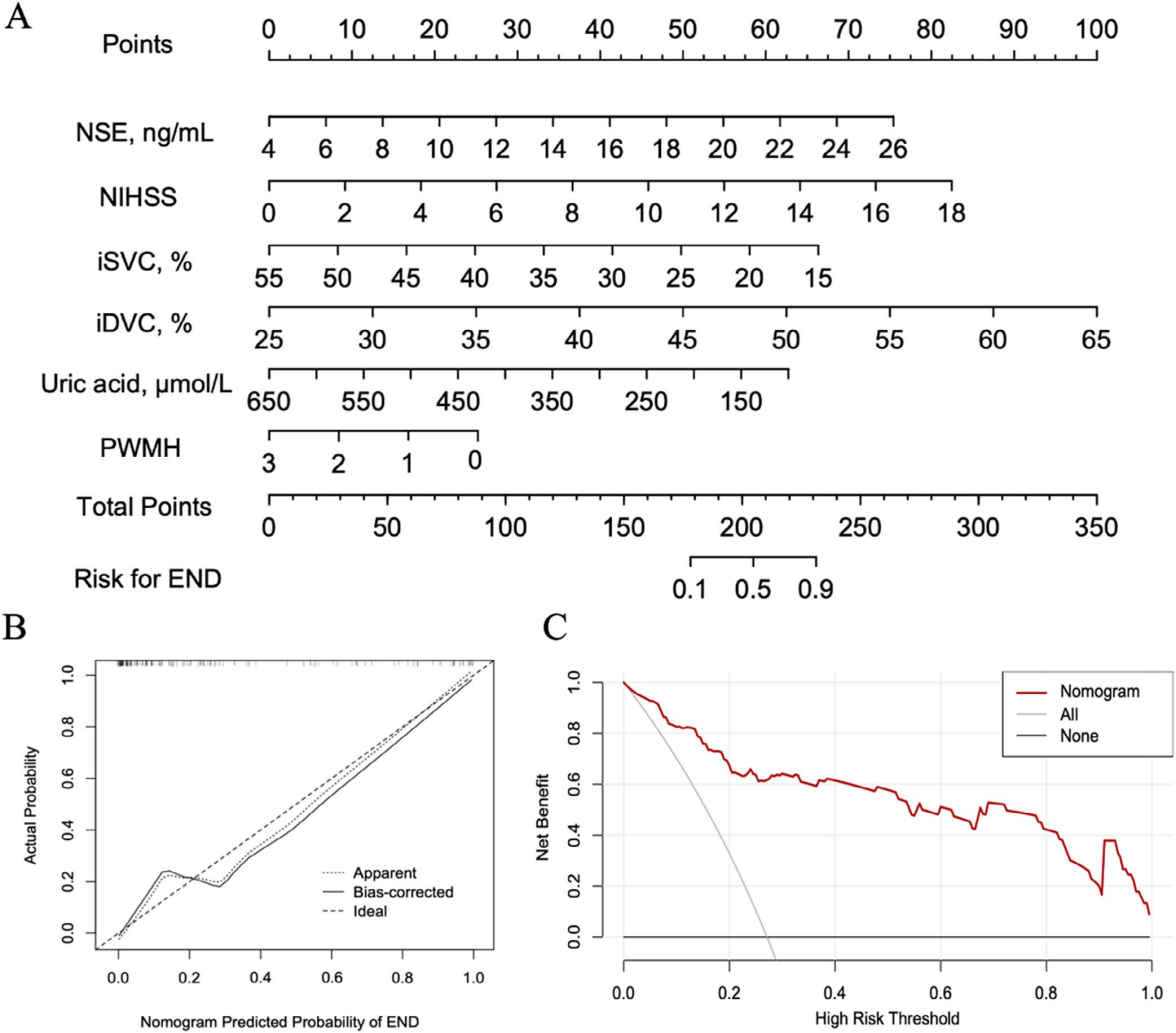
Developed nomogram for risk of END and its predictive performance and validation. (A) Nomogram predicting the incidence of END in our SSI cohort. (B) Calibration curves of the END nomogram prediction in our SSI cohort. The x‐axis shows the predicted END risk, while the y‐axis shows the actual diagnosed END. The diagonal dotted line depicts a perfect prediction by an ideal model. The solid line reflects the performance of the nomogram, where a closer fit to the diagonal dotted line indicates a better prediction. (C) Decision curve analysis for the END nomogram.

Based on the data obtained, a multivariate logistic regression analysis showed that serum NSE (OR 1.328, 95% CI: 1.113–1.584, *p* = 0.002), NIHSS (OR 1.461, 95% CI: 1.237–1.726, *p* < 0.001), iSVC (OR 0.871, 95% CI: 0.787–0.966, *p* = 0.009), iDVC (OR 1.229, 95% CI: 1.055–1.435, *p* = 0.008), serum uric acid (OR 0.991, 95% CI: 0.984–0.997, *p* = 0.004), and PWMH (OR 0.499, 95% CI: 0.257–0.968, *p* = 0.040) were independent predictors for END (Figure 5). The above independent predictors were then incorporated to develop a predictive nomogram, shown in Figure 6A.

### 3.3 Performance and Validation of the Nomogram

In our study cohort, the calibration curve of the END nomogram for SSI patients showed good agreement as seen in Figure 6B. The nomogram had an unadjusted C‐index for the END prediction nomogram of 0.922 (95% CI: 0.879–0.964) and a bootstrap‐corrected C‐index of 0.921, indicating good discrimination. Additionally, the Hosmer–Lemeshow test, *p* = 0.544, suggested that the model was of goodfit.

### 3.4 Clinical Utility

The decision curve analysis (DCA) for the END risk nomogram is presented in Figure 6C. The DCA showed that if the threshold probability was > 0.15, using the developed nomogram to predict the incidence of early neurological deterioration would provide more net benefit than either predictingall patients as END or predicting none of the tients as END.

### 3.5 Probability of Predicting END Based on the Nomogram Scores

The optimal cutoff value of the total nomogram scores was determined to be 203. At the optimal cutoff value, the nomogram had a sensitivity of 90%, specificity of 82%, positive predictive value of 94%, negative predictive value of 12%, positive likelihood ratio of 5.17, and a negative likelihood ratio of 0.12, as shown in Table 2.

**TABLE 2:** Accuracy of the prediction score of the nomogram for estimating the risk of END.

Variable	Value (95% CI)
AUC	0.92 (0.88–0.96)
Cutoff score	203
Sensitivity, %	0.90 (0.84–0.95)
Specificity, %	0.82 (0.67–0.93)
Positive predictive value, %	0.94 (0.88–0.98)
Negative predictive value, %	0.73 (0.58–0.85)
Positive likelihood ratio	5.17 (2.63–10.16)
Negative likelihood ratio	0.12 (0.07–0.20)

Abbreviations: AUC, the area under the ROC curve; CI, confidence interval; ROC, receiver operating characteristic.

## 4 Discussion

END is a common complication of SSI and is often caused by progressive tissue infarction, even in acute SSI patients who have received reperfusion therapy (i.e., thrombolysis). The poor prognosis of END makes it a major concern among SSI patients. Hence, improving precise identification and early detection of individuals with a high risk of END is necessary. In this study, combining multiscale biomarkers and OCTA‐derived metrics, we developed a novel nomogram that incorporated 4 easily available variables in routine clinical practice (NIHSS score, serum NSE and uric acid, PWMH by Fazekas scoring) with 2 OCTA‐derived retinal microvascular metrics in predicting END in SSI patients. Internal validation in the model revealed good discrimination and calibration ability with a C index of 0.92. The high C index in the internal validation showed that this nomogram can be extensively and accurately used on a large sample size. Moreover, the DCA showed that it added more benefit to predict END in our SSI cohort with a threshold exceeding 0.15, which demonstrated that the model is of clinical significance for decision‐making over a range of probability thresholds.

The multivariate regression analysis showed that predictors such as increased levels of serum NSE, higher NIHSS scores, decreased levels of serum uric acid, lower PWMH scores, and lower ipsilateral SVC and higher DVC density were independent predictors of END in our SSI cohort. As a result of these factors indicating diverse aspects of pathophysiological processes in acute subcortical infarction patients, the potential impacts on the development of END for acute SSI patients can be assessed in different ways, respectively.

The increasing homology between retinal and cerebral microcirculation makes retinal imaging useful in stroke cases. Using the OCTA, previous reports [8, 35, 36] showed that SSI patients had reduced retinal densities and sparser microvasculature compared to controls; these reports suggested that ischemic changes in the retina may be linked with ischemic changes in the brain. Here, we extended these findings and explored the retinal microvascular changes in SSI patients with and without END. We showed that SSI patients with END had lower SVC density compared to non‐END patients in eyes ipsilateral to the infarction. The SVC of the retina contains arterioles, venules, and capillaries [37]; this microvascular complex is sensitive to hypoxia and/or ischemia and is suggested to reflect the cerebral microcirculatory changes in cerebrovascular disorders [38]. In the retina, lower SVC density is indicative of tissue hypoxia and disturbed blood flow [39]. Similarly, END in SSI is suggested to be linked with cerebral hypoperfusion [40, 41]. Given the homology between the retina and the brain, reduced retinal microvascular density in the SVC of SSI patients with END may reflect corresponding microvascular abnormalities indicative of reduced microvascular impairment leading to ischemic damage in the cerebral microcirculation. Moreover, because these quantitative changes in the retinal microvasculature are associated with END, we also suggest that these lower retinal SVC densities may be a downstream effect of the occurrence of END in SSI in the retina and cerebral microcirculation. Taken together, the SVC is sensitive to retinal ischemia and reflects cerebral microcirculation changes. Given that END is associated with cerebral hypoperfusion, it is plausible to suggest that lower SVC density in END may reflect severe hypoperfusion.

A previous study using the OCTA showed SSI patients had lower DVC density compared to controls [35]. Here, we showed that SSI patients with END had a higher ipsilateral DVC density compared to patients without END. Recently, it has been found that microglia, as the main immune cells in the brain, play a role in ischemic stroke as proinflammatory cells in the acute phase and participate in the inflammatory cascade that exacerbates neurological damage [42]. Moreover, blood–brain barrier (BBB) disruption and leakage of plasma components are involved in the pathologic process of cerebral small vessel disease [43]. The DVC lies below the SVC, which is primarily found in the inner nuclear layer of the retina and consists of capillaries [44]. Importantly, the DVC is responsible for the venular circulation in the retina and is sensitive to inflammatory changes in the retina [45]. Some studies have illustrated that the DVC is sensitive to inflammation in many neurological diseases [46, 47]. We suggest that in acute SSI patients with END, inflammation may be active during the early phase, resulting in higher DVC density in ipsilateral eyes compared to contralateral eyes. Furthermore, we showed that higher DVC density in ipsilateral eyes was associated with a high risk of END in our SSI cohort. Given that higher DVC density reflects the inflammatory response in the acute phase of the pathophysiology of SSI, it is plausible to suggest that higher DVC density may be linked with the incidence of END in SSI. Further research with an expansion of the sample size is needed to test this hypothesis.

After brain injury, NSE could be released from the damaged neurons into the blood through an impaired BBB. Previous studies [48, 49] suggested that NSE can be used to evaluate microcirculation and neurological function in patients with ischemic stroke. We showed that increased serum levels of NSE are associated with the occurrence of END, suggesting that serum levels of NSE are an important factor for END in SSI patients.

Recently, few studies have explored the association between serum uric acid and the prognosis of ischemic stroke. Whether serum uric acid is a protective or destructive factor in ischemic stroke is still unclear. While some reports [50, 51] have shown that increased serum uric acid is linked with poor outcomes of ischemic stroke, others [52, 53] showed the opposite. Our study showed that lower levels of serum uric acid were an important risk factor for the occurrence of END in SSI. Our findings suggest that serum uric acid may be a useful marker to predict END and guide the individual treatment regimen for SSI patients with END risk.

Consistent with the results reported in previous studies [1, 3, 54, 55], our study found that SSI patients with higher NIHSS scores on admission are likely to have END. We suggest that higher NIHSS scores at admission may be an independent risk factor for the occurrence of END in SSI patients.

We showed that lower PWMH scores are an independent risk factor for the occurrence of END in our SSI cohort. On the one hand, PWMH is more likely to be influenced by long‐term and chronic hemodynamic insufficiency (hypoperfusion) [56]. It has been clarified in large clinical trials that acute ischemic stroke patients who received chronic remote ischemic conditioning were more likely to have favorable prognoses [5, 57, 58, 59]. Here, we speculate that patients who lack chronic cerebral hypoperfusion, that is, SSI patients with lower PWMH scores in our cohort, may have poorer compensatory ability for acute brain ischemia, making them more prone to experiencing severe neural damage during acute cerebral ischemic incidents. This hypothesis requires further validation through future in‐depth research.

On the other hand, this result highlights a different aspect of white matter pathology. PWMH is associated with SVD in the superficial white matter regions close to the ventricles, which are vulnerable to changes in blood–brain barrier permeability and cerebral fluid dynamics [60]. The association seen in our current study may suggest that PWMH may influence long‐term recovery trajectories rather than END. Advanced neuroimaging studies with extended follow‐up are needed in this field.

Our model demonstrated a higher prediction model for predicting END in SSI. A growing number of studies have shown the critical characteristics of stroke/SSI, such as vasculopathy (brain barrier damage and SVD) and neurodegeneration, manifesting in the retina and comparable to those detected in stroke/SSI [10, 61, 62, 63]. These studies have shown the key clinical findings and histopathological evidence in the retina of stroke/SSI. Common pathophysiological processes were reported in both the retina and brain of stroke/SSI patients. In our study, the inclusion of retinal microvasculature serving as a proxy to cerebral microcirculation with multiscale biomarkers may have played a role in the higher accuracy for predicting END in SSI. Given the homology between the retina and brain, our results suggest that ischemia (hypoperfusion) plays a vital role in END in SSI, and retinal vasculature may serve as a potential route to assess and detect it.

Several limitations need to be recognized. First, our study was based on a single‐center study with a relatively small sample size, which may introduce probable selection bias. Thus, a multicenter study with a large sample size is needed to validate our results. Second, not all clinical information, such as collateral circulation status, was included in this study; therefore, the role of other potential factors may need to be considered in future studies and/or models. More importantly, external validation is still required to further evaluate the performance of our nomogram predictive model. External validation using an independent and different cohort is necessary to further assess the generalizability of our model.

## 5 Conclusions

Our study developed and validated a nomogram‐based tool to predict high‐END risk individuals in SSI patients. This innovative nomogram exhibited commendable accuracy and discriminatory capabilities. This personalized risk evaluation instrument could aid clinicians in the early identification of SSI patients at a heightened risk of END, prompting timely medical interventions and ultimately contributing to an improved prognosis.

## Conflicts of Interest

The authors declare no conflicts of interest.

## Supporting information


Appendix S1.

## Data Availability

The raw data for the results of this study can be made available upon request from the corresponding author.
