# Supplementary material for: A Novel Nomogram Integrating Retinal Microvasculature and Clinical Indicators for Individualized Prediction of Early Neurological Deterioration in Single Subcortical Infarction
Source: CNS Neurosci Ther. 2025 Mar 12;31(3):e70337. doi: 10.1111/cns.70337 (PMC11903217; doi:10.1111/cns.70337)
Supplement: Supplementary file 1 — Appendix S1. [file CNS-31-e70337-s001.docx]

**Supplementary Material**

Reliability of Measurements of Neuroimaging Markers

Two trained neurologists (C.Y. and R.P.) were engaged in the MRI processing and measurements blinded to clinical information, and an experienced neurologist (B.W.) was consulted when disagreement occurred. The inter-rater reliability of measurements for each neuroimaging marker mentioned above was considered good to excellent: the intraclass correlation coefficients (ICC) for the lesion diameter and lesion slices were 0.85 and 0.88, respectively; the kappa-values for the severity of DWMH, PWMH were 0.75 and 0.80, and for the presence of sICH was 0.88.

OCTA Features Obtaining Details

The imaging tool (SS-OCTA; SVision Imaging, Henan, China. Version 2.1.016) contained a swept-source laser with a central wavelength of 1050nm and had a scan rate of 200,000 A-scans per second. The tool also had eye-tracking software to eliminate eye-motion artifacts. The axial resolution was 5 µm, lateral resolution was 13 µm and scan depth was 3mm. OCTA fundus images were obtained at the macula with a raster scan protocol of 384 horizontal B-scans that covered an area of 3 × 3 mm2 centered on the fovea. En face angiograms of the superficial vascular complex (SVC) and deep vascular complex (DVC) were generated by automatic segmentation. The segmentation between the SVC and the DVC was set in the inner two-thirds and outer one-third interface of ganglion cell layer and inner plexiform layer. The percentage of the SVC and DVC was obtained with an in-built algorithm in the OCTA tool in a 2.5mm diameter circular region centered on the fovea.

The OCTA data displayed in our study followed the OSCAR-IB quality criteria[1] and APOSTEL recommendation[2].

**Tables. Baseline comparisons between excluded and included populations**

|  | Not included | Included | P |
| --- | --- | --- | --- |
|  | 178 | 166 |  |
| Age, years | 56.93±10.29 | 56.72±10.40 | 0.847 |
| Males, n | 148 (83.15%) | 137 (82.53) | 0.993 |
| Smoking | 93 | 86 | 1 |
| NIHSS | 4 (1-5) | 4 (0-5) | 0.896 |
| Hypertension | 111 (62.36%) | 102 (61.45%) | 0.950 |
| Diabetes | 60 (33.71%) | 53 (31.93) | 0.813 |
| Hyperlipidemia | 45 (25.28%) | 41 (24.70%) | 1 |
| BMI | 24.76±3.16 | 24.69±3.15 | 0.836 |
| END | 48 (26.97%) | 45 (27.11) | 1 |

Data are n (%), mean (SD), or median (IQR).

NIHSS, National Institute of Health Stroke Scale score; BMI, BMI, body mass index; SBP, systolic blood pressure; DBP, diastolic blood pressure.

Reference

[1] Tewarie P, Balk L, Costello F, et al. The oscar-ib consensus criteria for retinal oct quality assessment. PLoS One[J]. 2012;7:e34823. <https://doi.org/10.1371/journal.pone.0034823>

[2] Aytulun A, Cruz-Herranz A, Aktas O, et al. Apostel 2.0 recommendations for reporting quantitative optical coherence tomography studies. Neurology[J]. 2021;97:68-79. <https://doi.org/10.1212/WNL.0000000000012125>
